# Production of Recombinant Hemoglobin in *Tremella fuciformis* Yeast-like Cells Through Selection of Hemoglobins and Secretion Elements

**DOI:** 10.3390/foods15173026

**Published:** 2026-08-27

**Authors:** Xiaoying Huang, Mei Hao, Yuxin Gou, Rui Li, Xuetuan Wei, Aimin Ma

**Affiliations:** College of Food Science and Technology, Huazhong Agricultural University, Wuhan 430070, China; 13720251911@163.com (X.H.); hmxxnlll@163.com (M.H.); 15934469396@163.com (Y.G.); lirui1232024@163.com (R.L.)

**Keywords:** *Tremella fuciformis*, yeast-like cells, recombinant hemoglobin, food biotechnology, secretion element

## Abstract

Hemoglobins have attracted increasing interest for their potential applications in alternative protein foods and food biotechnology, but the use of fungal yeast-like cells for recombinant hemoglobin production remains underexplored. In this study, *Tremella fuciformis* yeast-like cells (YLCs) were evaluated as a potential host system by screening candidate hemoglobins and secretion elements. Clover hemoglobin from *Trifolium subterraneum* (CHb), *Vitreoscilla* hemoglobin (VHb), and *Vigna unguiculata* leghemoglobin 2 (Lb2) were heterologously expressed in *T. fuciformis* YLCs. The estimated yields of CHb, VHb, and Lb2 were 32.00, 19.03, and 23.51 mg/L, respectively, with CHb showing the highest observed yield among the three tested hemoglobins. CHb was then used to evaluate the CysR signal peptide, α-mating factor prepro leader (Amf), and glucoamylase signal peptide (Gla). All three elements mediated CHb secretion, with Amf producing the highest extracellular CHb yield of 106.43 mg/L and total CHb production of 133.02 mg/L, approximately 4.16 times that of CHb without a secretion element. CysR and Gla produced extracellular CHb yields of 72.54 and 49.50 mg/L, respectively. These results identify CHb and Amf as a promising expression combination in *T. fuciformis* YLCs, but direct characterization of heme incorporation and functional properties is required before food applications can be evaluated.

## 1. Introduction

*Tremella fuciformis* is an important edible and medicinal fungus. During its yeast-like cell stage, it grows in a unicellular form that is suitable for liquid fermentation and possesses eukaryotic post-translational modification capabilities. These features make *T. fuciformis* yeast-like cells (YLCs) a promising expression platform for food-related recombinant protein production [1]. In recent years, advances in genetic manipulation techniques have expanded the use of *T. fuciformis* as a heterologous expression host, enabling the successful production of a variety of heterologous products, including natural compounds, vaccine antigens, and functional proteins [2,3,4]. However, current research has mainly focused on the construction, expression, and functional characterization of target products, whereas investigations into suitable target proteins and efficient secretory expression strategies remain limited.

Hemoglobins are heme-containing globin proteins that are widely distributed across diverse organisms [5]. With increasing interest in alternative protein foods, food fortification, and food biotechnology, hemoglobins have attracted attention for their nutritional value as well as their potential roles in color formation and flavor generation [6,7,8]. Previous studies have reported the successful expression of *Vitreoscilla* hemoglobin (VHb) in *T. fuciformis* [9]. Meanwhile, plant-derived hemoglobins have attracted increasing attention because of their potential applications in food fortification and meat substitute products. Among them, clover hemoglobin (CHb) from *Trifolium subterraneum* has been reported to exhibit desirable color stability and has been proposed as a promising ingredient for meat alternative products [10]. Therefore, VHb, CHb, and *Vigna unguiculata* leghemoglobin 2 (Lb2) were selected as candidate hemoglobins in this study to compare their expression characteristics in *T. fuciformis* YLCs and to identify a suitable hemoglobin for production in this system.

For recombinant proteins produced in a secreted form, release of the target protein into the culture medium facilitates downstream recovery and purification. Therefore, secretory expression is an important strategy for recombinant protein production [11,12]. In eukaryotic expression systems, signal peptides direct nascent proteins into the endoplasmic reticulum (ER) and initiate the secretory pathway. Consequently, the selection of signal peptides or secretion leaders can significantly influence protein translocation, processing, and overall secretion efficiency. Previous studies have demonstrated that different secretion elements can exhibit markedly different efficiencies for recombinant protein secretion in the same host system [13]. Therefore, screening secretion elements that are compatible with both the target protein and the host system has become an effective strategy for improving heterologous protein secretion. Nevertheless, comparative studies of secretion elements for heterologous protein production in *T. fuciformis* YLCs remain scarce.

Based on these considerations, *T. fuciformis* YLCs were employed as the expression host in this study. First, the expression characteristics of three candidate hemoglobins, CHb, VHb, and Lb2, were compared to identify a suitable hemoglobin for expression in *T. fuciformis* YLCs. Subsequently, the effects of three secretion elements, namely the CysR signal peptide, the α-mating factor prepro leader (Amf), and the glucoamylase signal peptide (Gla), on the secretory expression of the selected hemoglobin were evaluated. The present study extends previous work on heterologous expression in *T. fuciformis* by combining candidate hemoglobin comparison with subsequent secretion-element screening within the same host background, thereby evaluating recombinant hemoglobin production at both the target-protein and secretion-strategy levels. These findings provide an experimental basis for further optimization of *T. fuciformis* YLCs as a host for the production of recombinant hemoglobin and other food-related recombinant proteins.

## 2. Materials and Methods

### 2.1. Strains, Plasmids, Genes, and Reagents

The *T. fuciformis* yeast-like cell strain 32Y was preserved in the Microbiology Laboratory, College of Food Science and Technology, Huazhong Agricultural University. The strain was routinely cultivated and maintained on potato dextrose agar (PDA) medium (potato 200 g/L, glucose 20 g/L, and agar 20 g/L) at 25 °C. *Escherichia coli* DH5α (Tsingke Biotechnology Co., Ltd., Beijing, China) was used for plasmid propagation and cultured in Luria–Bertani (LB) medium (NaCl 10 g/L, yeast extract 5 g/L, tryptone 10 g/L, agar 15 g/L) at 37 °C. *Agrobacterium tumefaciens* EHA105 (Weidi Biotechnology Co., Ltd., Shanghai, China) was employed for genetic transformation of *T. fuciformis* YLCs and cultured in LB medium at 28 °C. Three hemoglobin genes from different biological sources were selected as candidate genes, including *chb* (GenBank protein accession no. GAU42437.1) from *Trifolium subterraneum*, *vgb* (GenBank protein accession no. AAA27585.1) from *Vitreoscilla*, and *lb2* (GenBank protein accession no. AAB65768.1) from *V. unguiculata*, encoding CHb, VHb, and Lb2, respectively. All genes were codon-optimized based on the preferred codon usage of *T. fuciformis* and chemically synthesized (Tsingke Biotechnology Co., Ltd., Beijing, China). The candidate secretion elements included the CysR signal peptide derived from a predicted cysteine-rich secreted protein of *T. fuciformis*, the *Saccharomyces cerevisiae* α-mating factor prepro leader (MFα prepro leader, Amf), and the glucoamylase signal peptide (Gla) from *Aspergillus niger*. CysR and Gla corresponded to the N-terminal signal peptide regions of their respective secreted proteins, whereas Amf represented the complete MFα prepro leader containing both the pre-region and pro-region. The cloning vector pMD18-T (TaKaRa Bio, Dalian, China) was used for TA cloning. The expression vector pGEH was derived from the binary vector pCAMBIA1302 (YRGene Biotechnology Co., Ltd., Changsha, China), in which the *T. fuciformis* glyceraldehyde-3-phosphate dehydrogenase promoter (*Ygpd*) drives the fused expression of the enhanced green fluorescent protein gene (*egfp*) and the hygromycin B resistance gene (*hyg*), serving as the reporter gene and selection marker, respectively. Restriction enzymes (TaKaRa Bio, Dalian, China) were used for vector construction. Unless otherwise stated, routine chemical reagents were purchased from Sinopharm Chemical Reagent Co., Ltd. (Shanghai, China). For expression analysis, recombinant strains were inoculated into CM medium (glucose 20 g/L, (NH_4_)_2_SO_4_ 1.32 g/L, MgSO_4_·7H_2_O 0.25 g/L, KH_2_PO_4_ 0.5 g/L, vitamin B1 0.2 mg/L, ZnSO_4_·7H_2_O 2 mg/L, CaCl_2_·2H_2_O 0.5 g/L) and cultivated at 25 °C with shaking at 150 r/min. After cultivation, cells and culture supernatants were collected separately for RT-qPCR-based transcript analysis, protein expression analysis, and evaluation of secretory expression. Induction medium (IM) contained KH_2_PO_4_ 3.0 g/L, NaH_2_PO_4_ 1.0 g/L, NH_4_Cl 1.0 g/L, MgSO_4_·7H_2_O 0.30 g/L, KCl 0.15 g/L, CaCl_2_ 0.01 g/L, FeSO_4_·7H_2_O 2.5 mg/L, glycerol 0.5% (*v*/*v*), glucose 10 mM, MES 40 mM, and acetosyringone 0.2 mM. IM was used for induction of *A. tumefaciens* virulence.

### 2.2. Construction of Expression Vectors

The primers used for vector construction and RT-qPCR analysis are listed in Table 1 and were synthesized by Tsingke Biotechnology Co., Ltd. (Beijing, China). Polymerase chain reaction (PCR) amplification was performed using 2× Taq Master Mix (Vazyme Biotech Co., Ltd., Nanjing, China) under the following conditions: initial denaturation at 95 °C for 2 min and 34 cycles of 95 °C for 20 s, 61 °C for 20 s, and 72 °C for 1 min, followed by a final extension at 72 °C for 5 min and holding at 12 °C. The recombinant hemoglobin constructs encoded a C-terminal 6× His tag introduced through the corresponding reverse primers.

#### 2.2.1. Construction of Hemoglobin Overexpression Vectors

Hemoglobin overexpression vectors were constructed using a stepwise cloning strategy. First, the *T. fuciformis Ygpd* promoter was amplified and inserted into the pMD18-T vector by TA cloning to generate the intermediate plasmid 18-T-Ygpd. The codon-optimized *chb*, *vgb*, and *lb2* genes were subsequently inserted into 18-T-Ygpd to obtain the corresponding intermediate expression constructs. The complete Ygpd-Hb expression cassettes were then amplified and directionally inserted into the pGEH expression vector, generating pGEH-Ygpd-chb, pGEH-Ygpd-vgb, and pGEH-Ygpd-lb2, respectively. All recombinant plasmids were verified by colony PCR and DNA sequencing (Tsingke Biotechnology Co., Ltd., Wuhan, China).

#### 2.2.2. Construction of Secretion Element Fusion Expression Vectors

CHb was selected as the common target protein for secretion element screening. The fusion sequences CysR-chb, Amf-vgb, and Gla-lb2 were chemically synthesized by Tsingke Biotechnology Co., Ltd. (Beijing, China). Each fusion fragment was amplified using the primers listed in Table 1 and inserted into the intermediate plasmid 18-T-Ygpd following the stepwise cloning strategy described above, generating 18-T-Ygpd-CysR-chb, 18-T-Ygpd-Amf-vgb, and 18-T-Ygpd-Gla-lb2. The complete Ygpd-secretion element-Hb expression cassettes were subsequently amplified and directionally inserted into the pGEH vector to obtain pGEH-Ygpd-CysR-chb, pGEH-Ygpd-Amf-vgb, and pGEH-Ygpd-Gla-lb2.

To generate secretion element fusion constructs containing the same target hemoglobin, pGEH-Ygpd-Amf-vgb and pGEH-Ygpd-Gla-lb2 were linearized using *Apa*I and *Kpn*I. The *chb* coding sequence was amplified using the primer pairs chb-F(amf-chb)/gene-R and chb-F(gla-chb)/gene-R, respectively, with homologous sequences corresponding to the ends of the linearized vectors. The amplified *chb* fragments were inserted into the corresponding linearized vectors by In-Fusion cloning, thereby replacing *vgb* and *lb2* and generating pGEH-Ygpd-Amf-chb and pGEH-Ygpd-Gla-chb, respectively. All intermediate and final recombinant plasmids were verified by colony PCR and DNA sequencing.

### 2.3. Agrobacterium-Mediated Transformation and Transformant Screening

Recombinant expression vectors were introduced into competent *A. tumefaciens* EHA105 cells using the freeze–thaw method according to the manufacturer’s instructions. The ATMT procedure for *T. fuciformis* YLCs was adapted from the method described by Wang et al. [14], with modifications. Briefly, *A. tumefaciens* harboring recombinant plasmids was cultured in LB medium supplemented with kanamycin (50 mg/L; Sigma-Aldrich, St. Louis, MO, USA) and rifampicin (50 mg/L; Sigma-Aldrich, St. Louis, MO, USA) until the optical density at 600 nm (OD_600_) reached 0.6–0.8. Cells were harvested by centrifugation and resuspended in IM to an OD_600_ of 0.15, followed by induction at 28 °C for 6 h. The induced *A. tumefaciens* suspension was then mixed with a suspension of YLCs (1 × 10^7^ CFU/mL) at a 1:1 volume ratio and spread onto IM agar plates covered with sterilized mixed cellulose ester membranes (0.45 μm pore size, 80 mm diameter; Shanghai Xingya, Shanghai, China). Co-cultivation was carried out at 25 °C for 3 d. Following co-cultivation, the membranes were transferred onto PDA selection plates supplemented with cefotaxime (200 mg/L; Sigma-Aldrich, St. Louis, MO, USA) and hygromycin B (50 mg/L; Yeasen Biotechnology (Shanghai) Co., Ltd., Shanghai, China). Stable transformants were obtained after three successive rounds of antibiotic selection. Genomic DNA was extracted from the transformants following the CTAB–phenol/chloroform method used by Wang et al. [14]. Transformant cells were ground into a fine powder in liquid nitrogen and incubated in CTAB extraction buffer containing 1% (*w*/*v*) CTAB, 100 mM Tris-HCl (pH 8.0), 1 M NaCl, and 20 mM EDTA at 70 °C for 30 min. The lysate was extracted with an equal volume of phenol/chloroform/isoamyl alcohol (25:24:1, *v*/*v*/*v*), and genomic DNA was precipitated from the aqueous phase with isopropanol, washed with 75% ethanol, and dissolved in 1× TE buffer. The presence of the *egfp–hyg* fusion fragment in the transformants was verified by PCR using genomic DNA as the template and the primer pair EH-F/EH-R listed in Table 1. PCR was performed under the conditions described in Section 2.2, and the products were analyzed by agarose gel electrophoresis. Expression of enhanced green fluorescent protein (eGFP) was examined using a Ti-S fluorescence microscope (Nikon, Tokyo, Japan). Images were acquired under identical exposure settings for wild-type and recombinant strains, and scale bars are indicated in the corresponding figure caption. Transformants confirmed by both PCR and eGFP fluorescence analyses were selected for subsequent expression studies.

### 2.4. Reverse Transcription Quantitative PCR Analysis

Reverse transcription quantitative PCR (RT-qPCR) was performed to determine the relative transcript abundance of the target genes. Total RNA was extracted from wild-type and recombinant strains using TRIzol reagent (TaKaRa Bio, Dalian, China). First-strand cDNA was synthesized using the PrimeScript™ RT Reagent Kit with gDNA Eraser (TaKaRa Bio, Dalian, China) to eliminate genomic DNA contamination. The *β-tubulin* gene was used as the reference gene. RT-qPCR was performed using TB Green Premix Ex Taq™ II (TaKaRa Bio, Dalian, China) on a QuantStudio™ 3 Real-Time PCR System (Applied Biosystems, Foster City, CA, USA). Primer sequences are listed in Table 1. The RT-qPCR program consisted of an initial denaturation at 95 °C for 10 min, followed by 40 cycles of 95 °C for 20 s, 60 °C for 20 s, and 72 °C for 20 s. Melting curve analysis was performed after amplification to verify product specificity. For the CHb-, VHb-, and Lb2-expressing strains, ΔCt values were calculated as Ct (target gene) − Ct (*β-tubulin*) and presented directly. For comparison of *chb* transcript abundance among strains carrying different secretion elements, ΔCt values were calculated as Ct (*chb*) − Ct (*β-tubulin*) and compared directly because the same *chb*-specific primer pair and reference gene were used for all constructs. Three independent transformants were analyzed for each construct, and each sample was measured in triplicate (technical replicates).

### 2.5. Protein Expression and Secretion Analysis

Recombinant strains were cultivated for 6 d in CM medium supplemented with 2.5 mM 5-aminolevulinic acid hydrochloride (ALA; Shanghai Macklin Biochemical Co., Ltd., Shanghai, China), which was added to support heme biosynthesis. For intracellular protein extraction, cell pellets were resuspended in protein extraction buffer containing 100 mM Tris-HCl (pH 7.5) and 300 mM NaCl, and then disrupted with glass beads (2–3 mm; Jiqi Crafts Business Department, Quanzhou, China) by vortexing for 15 s followed by cooling on ice for 45 s; this process was repeated 10 times. Cell debris was removed by centrifugation at 12,400× *g* for 20 min at 4 °C, and the clarified lysates were collected for protein analysis. Before TCA precipitation, aliquots of the clarified intracellular lysates and culture supernatants were used to determine total protein concentrations using a BCA Protein Assay Kit (Vazyme Biotech Co., Ltd., Nanjing, China) according to the manufacturer’s instructions. Separate aliquots were used for subsequent TCA precipitation and SDS-PAGE analysis. For protein precipitation and concentration, neat trichloroacetic acid (TCA; Sinopharm Chemical Reagent Co., Ltd., Shanghai, China) was melted at 65 °C, and 100 μL of the molten TCA was added to 900 μL of protein sample. The mixture was incubated at 4 °C for 30 min and then centrifuged at 8600× *g* for 10 min to collect the precipitated proteins. For secretion element fusion strains, culture supernatants were concentrated using the same TCA precipitation procedure and used for secreted protein analysis, while intracellular proteins were extracted and analyzed in parallel. Protein samples were separated by 15% sodium dodecyl sulfate-polyacrylamide gel electrophoresis (SDS-PAGE) using gels prepared with a commercial gel preparation kit (Vazyme Biotech Co., Ltd., Nanjing, China) according to the manufacturer’s instructions. A protein molecular weight marker (Vazyme Biotech Co., Ltd., Nanjing, China) was included, and protein bands were visualized using Coomassie Brilliant Blue Fast Staining Solution (BL607A; Biosharp Life Sciences, Hefei, China). Band intensities were quantified using ImageJ software 1.54g (National Institutes of Health, Bethesda, MD, USA). Target protein yield was estimated by multiplying the proportion of target-band intensity relative to the total lane intensity by the corresponding total protein concentration determined by BCA before TCA precipitation. For quantitative protein analyses, a selected transformant from each construct was independently cultured three times, and the three independent cultures were treated as biological replicates. For each independent culture, total CHb production was calculated as the sum of intracellular and extracellular CHb yields, whereas extracellular CHb proportion was calculated as the percentage of extracellular CHb yield relative to total CHb production.

### 2.6. Statistical Analysis

Statistical analyses were conducted using GraphPad Prism 8.0.2 software (GraphPad Software, San Diego, CA, USA). Data are presented as mean ± standard deviation (SD), unless otherwise indicated. Differences among the CysR, Amf, and Gla groups were evaluated by one-way analysis of variance (one-way ANOVA) followed by Tukey’s multiple comparison test. For quantitative protein analyses, independent cultures of a selected transformant were treated as biological replicates. Comparisons of total CHb production between the secretion-element groups and the CHb-only control were evaluated by one-way ANOVA followed by Dunnett’s multiple comparison test. Differences were considered statistically significant at *p* < 0.05.

## 3. Results

### 3.1. Construction and Verification of Expression Vectors

**Figure 1:**
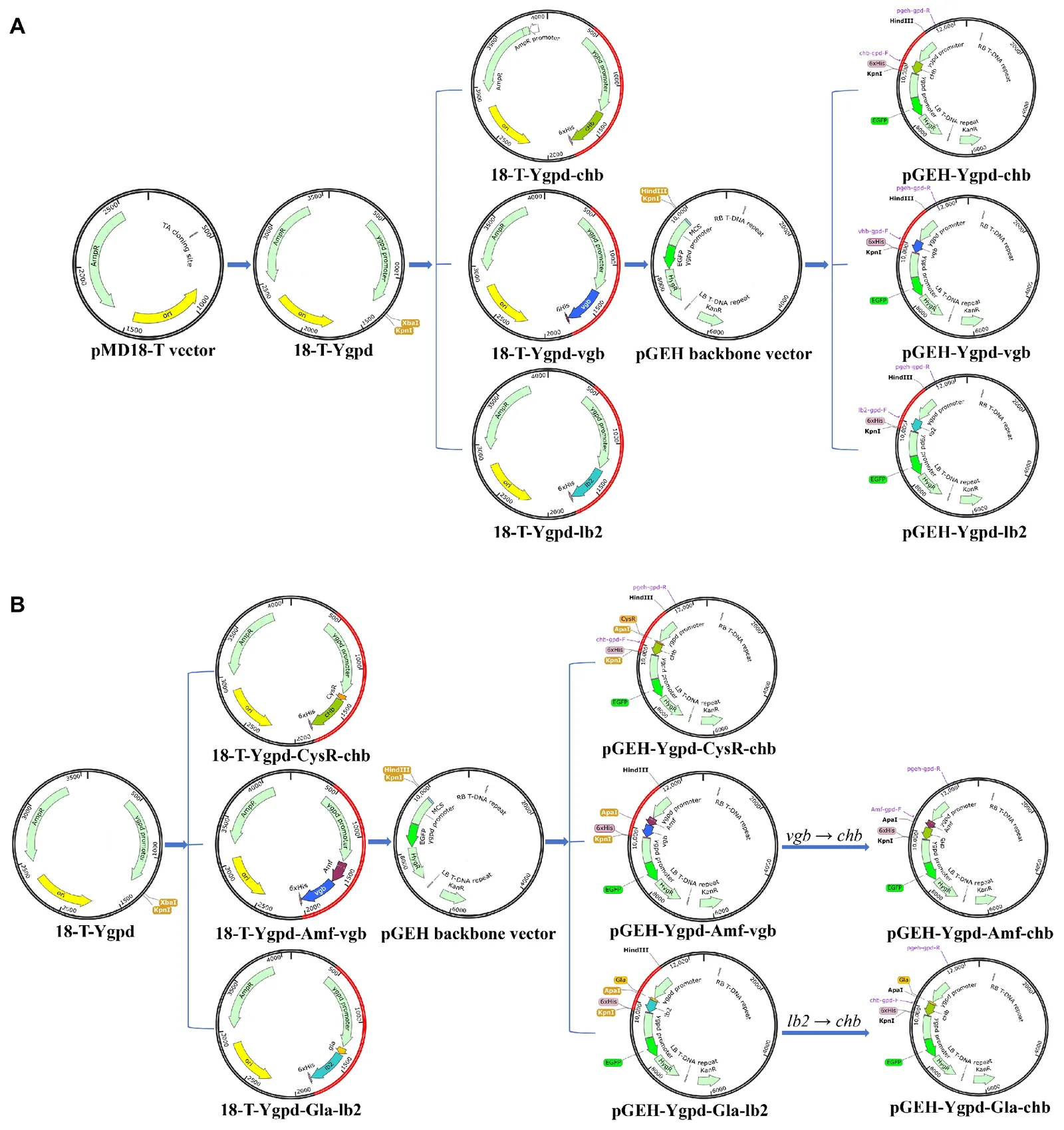
Construction strategies for hemoglobin expression vectors and secretion element fusion expression vectors. (**A**) Construction of hemoglobin expression vectors. Red arcs indicate the complete expression cassettes amplified from the corresponding 18-T-Ygpd intermediate plasmids and subcloned into the pGEH backbone through *Hin*dIII/*Kpn*I digestion and ligation. (**B**) Construction of secretion element fusion expression vectors. The chemically synthesized CysR-chb, Amf-vgb, and Gla-lb2 fusion fragments were introduced into 18-T-Ygpd, and the corresponding expression cassettes were subsequently transferred into pGEH using the same downstream cloning strategy as described in panel A. pGEH-Ygpd-CysR-chb, pGEH-Ygpd-Amf-vgb, and pGEH-Ygpd-Gla-lb2 were constructed in parallel. Because pGEH-Ygpd-CysR-chb already contained *chb*, it was used directly for secretion element screening. In the Amf and Gla constructs, *vgb* and *lb2* were subsequently replaced with *chb* by In-Fusion cloning following *Apa*I/*Kpn*I linearization, respectively.

**Figure 2:**
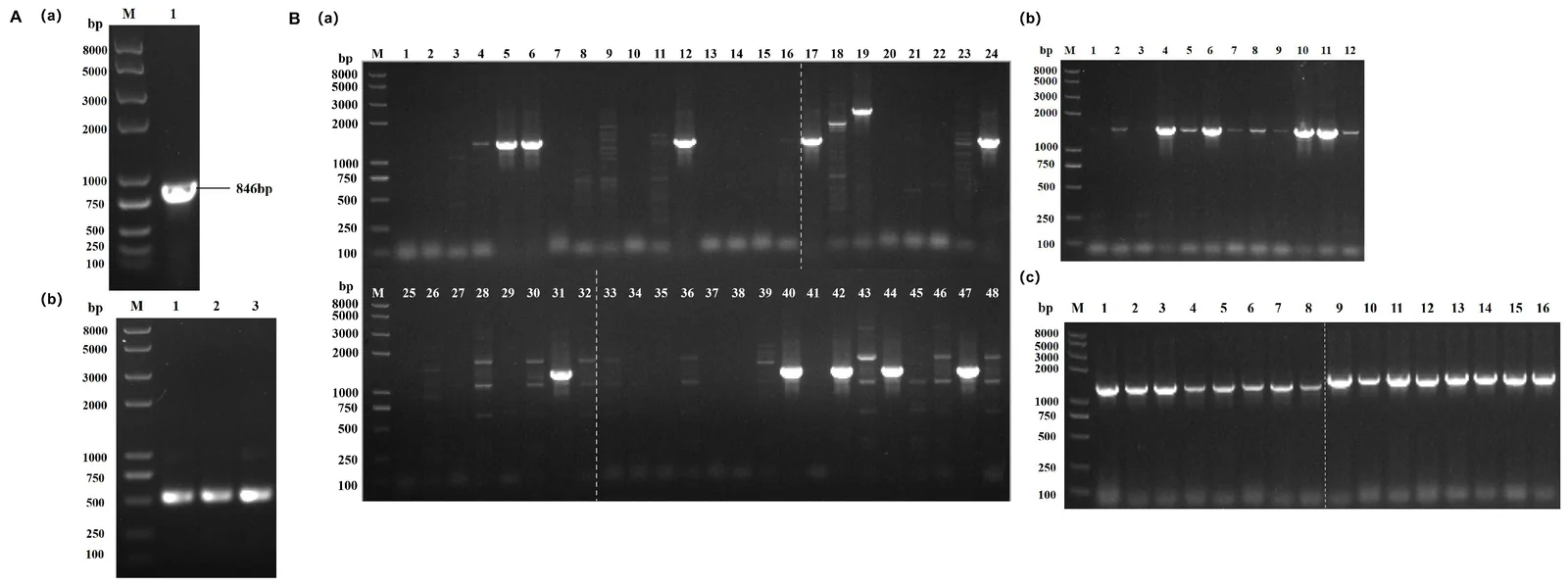
PCR amplification of target fragments and colony PCR screening of recombinant expression vectors. (**A**) PCR amplification of target fragments. (**a**) Amplification of the *Ygpd* promoter. Lane M, Trans 2K Plus II DNA Marker (TransGen Biotech, Beijing, China); Lane 1, *Ygpd*. (**b**) Amplification of candidate hemoglobin genes. Lane M, Trans 2K Plus II DNA Marker; Lane 1, *chb*; Lane 2, *vgb*; Lane 3, *lb2*. (**B**) Colony PCR screening of recombinant expression vectors. Colony PCR was performed using construct-specific forward primers: chb-gpd-F for pGEH-Ygpd-chb, pGEH-Ygpd-CysR-chb, and pGEH-Ygpd-Gla-chb; vhb-gpd-F for pGEH-Ygpd-vgb; lb2-gpd-F for pGEH-Ygpd-lb2; and Amf-gpd-F for pGEH-Ygpd-Amf-chb. All forward primers were paired with the common reverse primer pgeh-gpd-R. (**a**) Colony PCR screening of hemoglobin expression vectors. Lane M, Trans 2K Plus II DNA Marker; Lanes 1–16, candidate *E. coli* clones screened for pGEH-Ygpd-*chb*; Lanes 17–32, candidate *E. coli* clones screened for pGEH-Ygpd-vgb; Lanes 33–48, candidate *E. coli* clones screened for pGEH-Ygpd-lb2. (**b**) Colony PCR screening of the secretion element fusion expression vector pGEH-Ygpd-CysR-chb. Lane M, Trans 2K Plus II DNA Marker; Lanes 1–12, candidate *E. coli* clones screened for pGEH-Ygpd-CysR-chb. (**c**) Colony PCR screening of secretion element fusion expression vectors. Lane M, Trans 2K Plus II DNA Marker; Lanes 1–8, candidate *E. coli* clones screened for pGEH-Ygpd-Amf-chb; Lanes 9–16, candidate *E. coli* clones screened for pGEH-Ygpd-Gla-chb. PCR, polymerase chain reaction.

The construction strategies for the hemoglobin expression vectors and secretion element fusion expression vectors are illustrated in Figure 1. The *T. fuciformis Ygpd* promoter and the candidate hemoglobin genes *chb*, *vgb*, and *lb2* were amplified by PCR, yielding fragments of approximately 846, 441, 438, and 435 bp, respectively, consistent with their expected sizes (Figure 2A). Colony PCR screening identified candidate positive *E. coli* clones carrying pGEH-Ygpd-chb, pGEH-Ygpd-vgb, and pGEH-Ygpd-lb2, with amplicons of approximately 1430, 1407, and 1398 bp, respectively (Figure 2B(a)). DNA sequencing of the selected clones confirmed that the target fragments had been inserted at the intended sites and that the inserted sequences matched the designed constructs, verifying the successful construction of the three hemoglobin expression vectors.

Following transformation of *T. fuciformis* YLCs with these expression vectors and comparative evaluation of hemoglobin expression, CHb was selected as the target protein for secretion element screening. The secretion element fusion expression vectors pGEH-Ygpd-CysR-chb, pGEH-Ygpd-Amf-chb, and pGEH-Ygpd-Gla-chb were subsequently constructed. Colony PCR screening yielded amplicons of approximately 1493, 1361, and 1508 bp for the three vectors, respectively (Figure 2B(b,c)). DNA sequencing further confirmed that the inserted sequences matched the designed constructs, verifying the successful construction of all three secretion element fusion expression vectors.

### 3.2. PCR and eGFP Verification of Transformants

**Figure 3:**
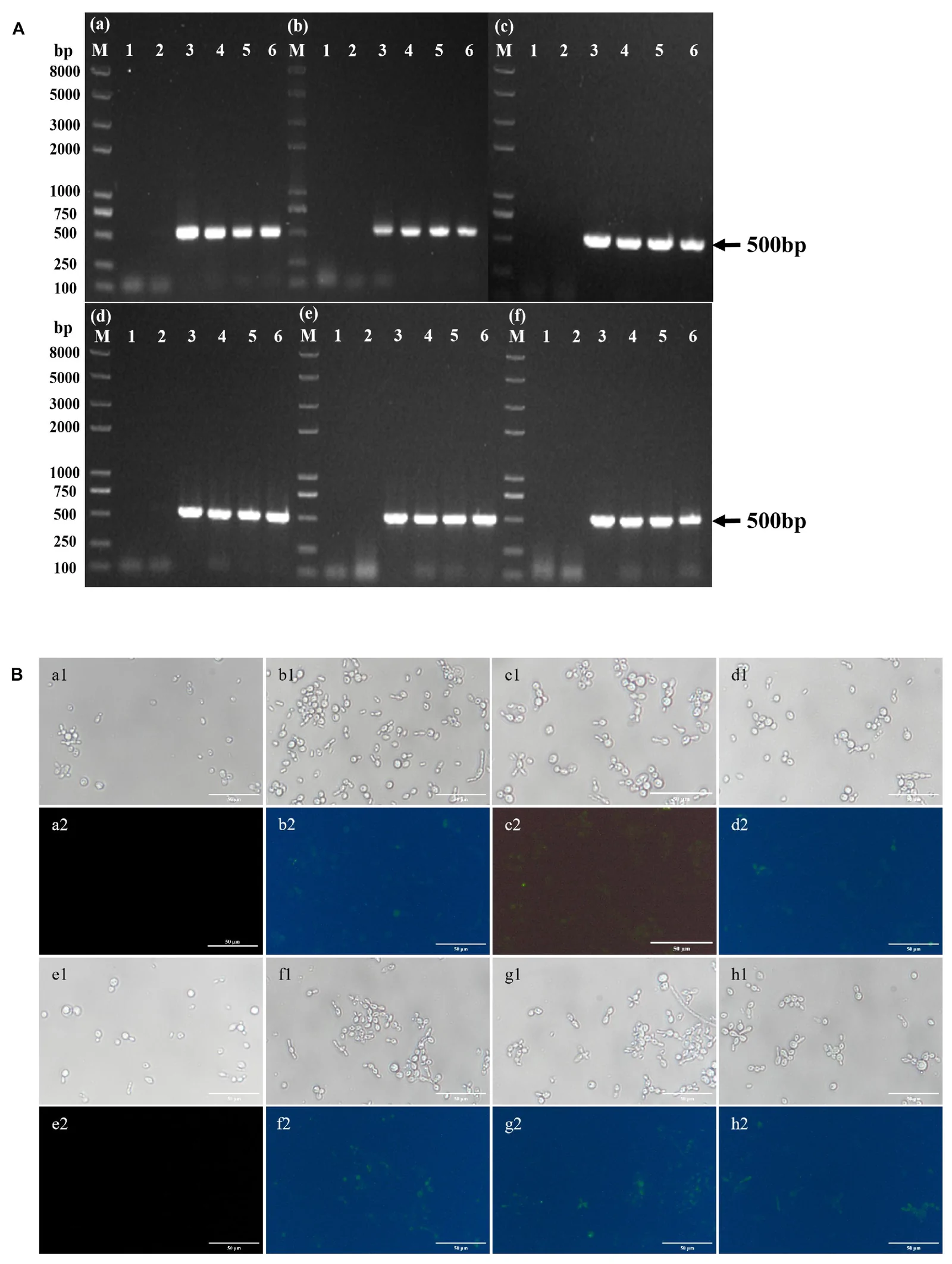
PCR and eGFP verification of *T. fuciformis* transformants. (**A**) Transformants were verified using the primer pair EH-F/EH-R. Lane M, Trans 2K Plus II DNA Marker; Lane 1, wild-type strain (WT); Lane 2, no-template control (NTC); Lane 3, recombinant expression vector; Lanes 4–6, three independent transformants. (**a**) pGEH-Ygpd-chb; (**b**) pGEH-Ygpd-vgb; (**c**) pGEH-Ygpd-lb2; (**d**) pGEH-Ygpd-CysR-chb; (**e**) pGEH-Ygpd-Amf-chb; (**f**) pGEH-Ygpd-Gla-chb. (**B**) eGFP fluorescence analysis of transformants. Panels (**a1**,**e1**) show bright-field images of the wild-type strain, while panels (**a2**,**e2**) show the corresponding eGFP fluorescence images. Panels (**b1**–**d1**,**f1**–**h1**) show bright-field images of the CHb, VHb, Lb2, CysR-CHb, Amf-CHb, and Gla-CHb transformants, respectively. Panels (**b2**–**d2**,**f2**–**h2**) show the corresponding eGFP fluorescence images. Scale bar: 50 μm. PCR, polymerase chain reaction; eGFP, enhanced green fluorescent protein; WT, wild type; NTC, no-template control.

Stable transformants were obtained after successive rounds of antibiotic selection. PCR analysis revealed that all tested transformants generated specific amplification products of approximately 500 bp, whereas no corresponding products were detected in the wild-type strain or the negative control (Figure 3A). These results indicated that the foreign expression cassettes had been successfully integrated into the *T. fuciformis* genome. Fluorescence microscopy showed detectable eGFP fluorescence signals in all tested transformants, whereas no corresponding fluorescence signal was observed in the wild-type strain (Figure 3B). Collectively, these results confirmed that the recombinant expression vectors had been successfully introduced into *T. fuciformis* YLCs and stably integrated into the genome.

### 3.3. Expression Screening of Candidate Hemoglobins

**Figure 4:**
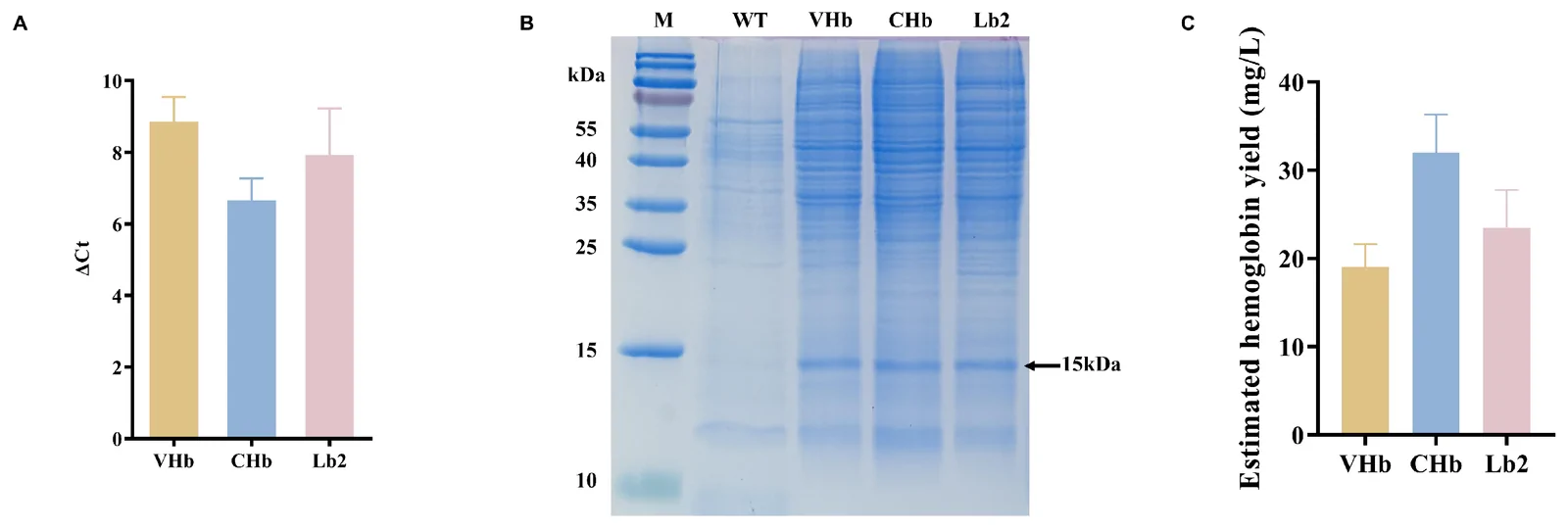
Expression analysis of hemoglobins from different sources in *T. fuciformis* YLCs. (**A**) ΔCt values of the three heterologous hemoglobin transcripts. ΔCt was calculated as Ct (target gene) − Ct (*β-tubulin*). Data are presented as mean ± SD from three independent transformants (*n* = 3 biological replicates), with each sample measured in triplicate. Lower ΔCt values indicate higher transcript abundance relative to β-tubulin within the corresponding RT-qPCR assay. (**B**) SDS-PAGE analysis of hemoglobin expression in *T. fuciformis* YLCs. Lane M, protein molecular weight marker; Lane WT, wild-type strain; Lane VHb, VHb transformant; Lane CHb, CHb transformant; Lane Lb2, Lb2 transformant. The arrow indicates the band at approximately 15 kDa, consistent with the expected molecular-weight region of the recombinant hemoglobins. (**C**) Comparison of estimated recombinant hemoglobin yields. Data are presented as mean ± SD from three independent cultures of a selected transformant for each hemoglobin construct (*n* = 3 biological replicates). SDS-PAGE, sodium dodecyl sulfate–polyacrylamide gel electrophoresis; CHb, clover hemoglobin; VHb, *Vitreoscilla* hemoglobin; Lb2, leghemoglobin 2; WT, wild type; SD, standard deviation.

RT-qPCR detected the corresponding heterologous hemoglobin transcripts in the CHb-, VHb-, and Lb2-expressing strains, and the ΔCt values normalized to *β-tubulin* are shown in Figure 4A. SDS-PAGE analysis revealed distinct protein bands at approximately 15 kDa, consistent with the expected molecular weights of the recombinant hemoglobins (Figure 4B). Protein yields were subsequently estimated based on densitometric analysis of these bands combined with total protein concentrations. The estimated yields of CHb, VHb, and Lb2 were 32.00 mg/L, 19.03 mg/L, and 23.51 mg/L, respectively, with CHb showing the highest observed yield among the three tested hemoglobins (Figure 4C). Based on the highest observed estimated protein yield among the three tested hemoglobins, CHb was selected as the target hemoglobin for subsequent secretion element screening and secretory expression studies.

### 3.4. Effects of Different Secretion Elements on CHb Production and Secretion

**Figure 5:**
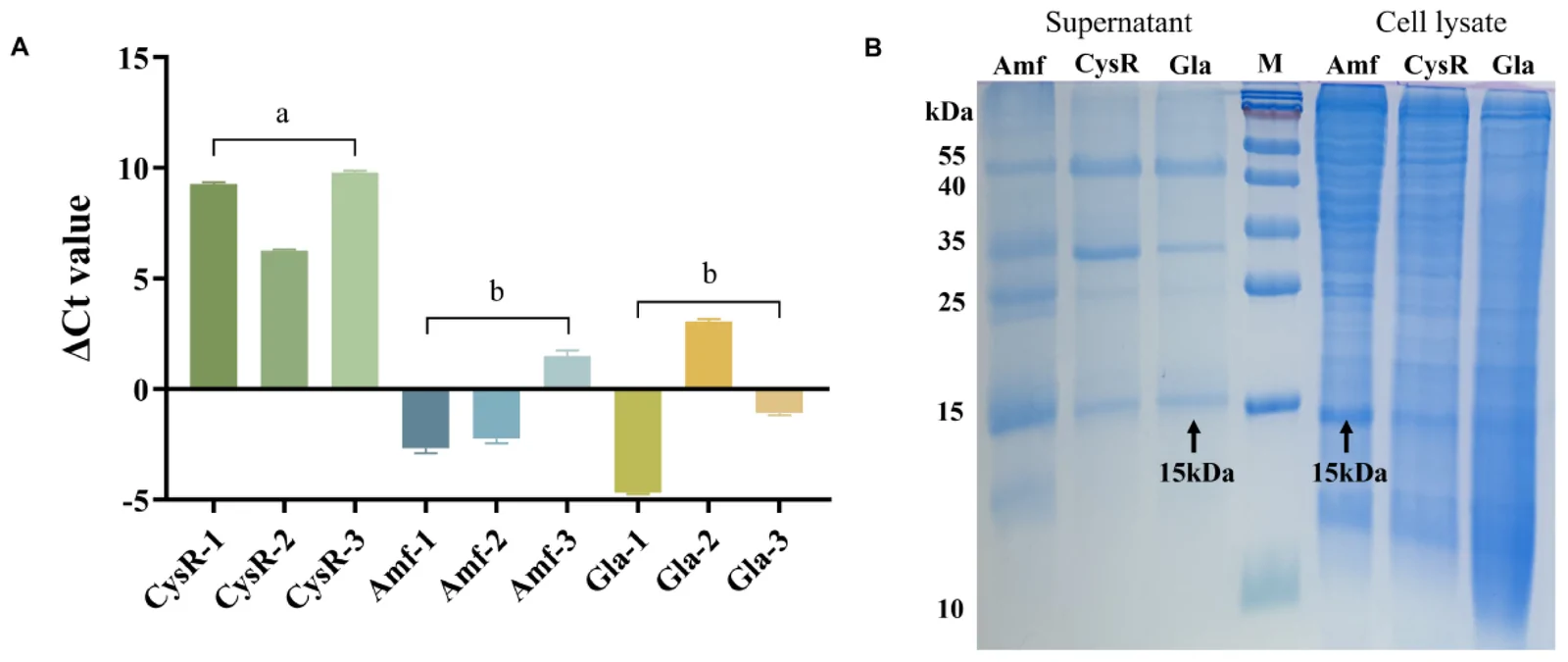
CHb transcript abundance and secretory expression in transformants carrying different secretion elements. (**A**) Analysis of ΔCt values in transformants carrying different secretion elements. ΔCt values were calculated as Ct (*chb*) − Ct (*β-tubulin*), with lower ΔCt values indicating higher *chb* transcript abundance relative to *β-tubulin*. CysR-1 to CysR-3, Amf-1 to Amf-3, and Gla-1 to Gla-3 represent three independent transformants of each construct. Each bar represents the mean ± SD of three technical RT-qPCR replicates for one independent transformant. Statistical comparisons among the CysR, Amf, and Gla groups were performed using the three independent transformants as biological replicates (*n* = 3). Different letters indicate significant differences among groups (*p* < 0.05). (**B**) SDS-PAGE analysis of CHb secretory expression mediated by different secretion elements. Lanes 1–3 represent culture supernatant samples corresponding to Amf-CHb, CysR-CHb, and Gla-CHb, respectively. Lane M, protein molecular weight marker. Lanes 4–6 represent cell lysate samples corresponding to Amf-CHb, CysR-CHb, and Gla-CHb, respectively. The arrows indicate bands at approximately 15 kDa, consistent with the expected molecular weight of CHb. ΔCt, Delta threshold cycle; CHb, clover hemoglobin; SDS-PAGE, sodium dodecyl sulfate-polyacrylamide gel electrophoresis.

**Figure 6:**
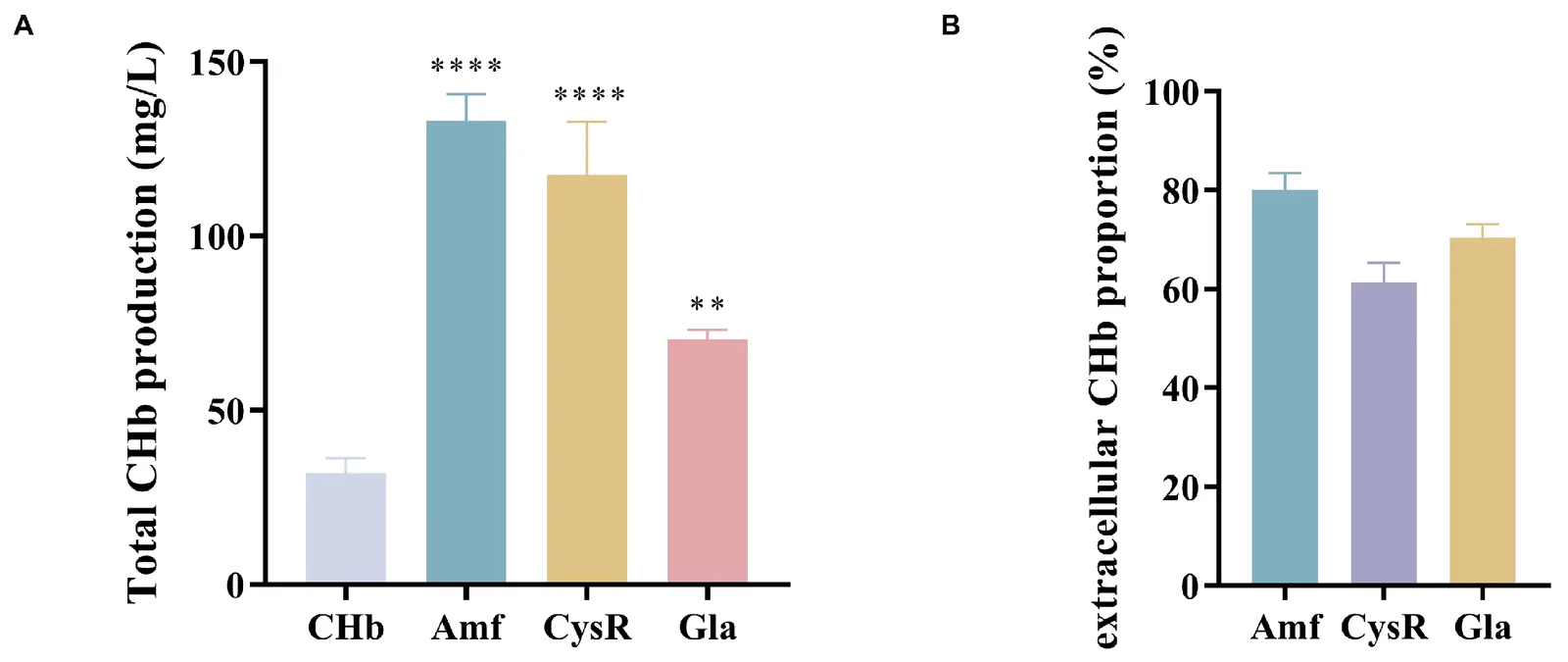
Analysis of total CHb production and extracellular CHb proportion with different secretion elements. (**A**) Comparison of total CHb production in different secretion element expression systems. CHb represents the transformant expressing CHb without a fused secretion element. Total CHb production was calculated as the sum of intracellular and extracellular CHb yields. Data are presented as mean ± SD from three independent cultures of a selected transformant for each construct (*n* = 3 biological replicates). **, *p* < 0.01; ****, *p* < 0.0001. (**B**) Comparison of extracellular CHb proportions among transformants carrying different secretion elements. The extracellular CHb proportion was calculated as the percentage of extracellular CHb relative to total CHb production. The extracellular CHb proportion was calculated independently for each culture, and data are presented as mean ± SD from three independent cultures of a selected transformant for each secretion-element construct (*n* = 3 biological replicates).

RT-qPCR analysis revealed significant differences in ΔCt values among the CysR-CHb, Amf-CHb, and Gla-CHb groups (Figure 5A). The mean ΔCt values were 8.44, −1.14, and −0.90 for CysR-CHb, Amf-CHb, and Gla-CHb, respectively. The ΔCt value of CysR-CHb was 9.58 and 9.34 cycles higher than those of Amf-CHb and Gla-CHb, respectively, and both differences were significant, whereas Amf-CHb and Gla-CHb differed by only 0.24 cycles, with no significant difference between them. These results indicated lower steady-state CHb transcript abundance in the CysR-CHb group. SDS-PAGE analysis showed protein bands at approximately 15 kDa, consistent with the expected molecular weight of CHb, in the culture supernatants of transformants carrying the three secretion elements (Figure 5B). Among them, the Amf-CHb group showed the strongest target protein band, followed by CysR-CHb, whereas Gla-CHb displayed the weakest signal. CHb bands were also detected in the cell lysate samples from all three groups. Quantitative analysis was subsequently performed to determine extracellular and intracellular CHb yields, total CHb production, and extracellular CHb proportion (Figure 6). No detectable CHb band was observed in the culture supernatant of the CHb-only transformant under the same analytical conditions (Figure S1); therefore, its measurable CHb production was represented by its intracellular CHb yield of 32.00 mg/L. The CHb-only data shown in Figure 6A were derived from the same measurements presented for CHb in Figure 4C and were reused here as the no-secretion-element control. Total CHb production was 133.02, 117.55, and 70.33 mg/L for Amf, CysR, and Gla, respectively. Compared with CysR, total CHb production was 15.47 mg/L higher for Amf and 47.22 mg/L lower for Gla. The total CHb production obtained with Amf was approximately 4.16 times the measurable CHb production of the CHb-only transformant (32.00 mg/L). For extracellular CHb production, Amf, CysR, and Gla produced extracellular CHb yields of 106.43 mg/L, 72.54 mg/L, and 49.50 mg/L, respectively. The observed extracellular CHb proportion was highest for Amf (80.0%), followed by Gla and CysR (Figure 6B). Collectively, these results identified Amf as the most effective of the three tested secretion elements for CHb secretory production in *T. fuciformis* YLCs.

## 4. Discussion

This study compared the heterologous production of three candidate hemoglobins, CHb, VHb, and Lb2, in *T. fuciformis* yeast-like cells. Although all three hemoglobin genes were driven by the same *Ygpd* promoter and codon-optimized according to the codon usage preference of *T. fuciformis*, the three constructs exhibited different estimated recombinant protein yields. Similar variation has been reported in other yeast-based hemoglobin production systems. Xue et al. evaluated the production of multiple hemoglobins and myoglobins in an engineered *S. cerevisiae* system and observed substantial differences in globin yields, with soybean hemoglobin reaching 108.2 ± 3.5 mg/L, whereas clover hemoglobin reached only 13.7 ± 0.5 mg/L [15]. These findings further indicate that different globins can exhibit markedly different production levels within the same yeast production system, highlighting the practical value of experimentally screening candidate hemoglobins for a given production system. For recombinant hemoglobins, globin synthesis alone does not ensure the formation of mature hemoglobin; heme incorporation and subsequent maturation are also important steps in this process [16]. In *Pichia pastoris*, enhancement of intracellular heme supply increased the heme-binding ratios of soybean hemoglobin and porcine myoglobin to 79.47% and 60.02%, respectively, corresponding to 3.67- and 2.87-fold increases relative to the corresponding controls, while increasing their protein yields by 55.43% and 52.01%, respectively [17]. Taken together, these observations suggest that, in addition to globin production itself, heme availability and incorporation may influence the formation and accumulation of mature hemoglobin. Although ALA was supplemented during cultivation to support heme biosynthesis, heme incorporation into the recombinant globins was not directly determined. Therefore, the protein yields reported in this study represent estimates of recombinant globin production and should not be interpreted as direct quantification of holohemoglobin production. The C-terminal 6× His tag present in the recombinant constructs may facilitate affinity purification in future studies, enabling direct characterization of heme incorporation and functional properties.

This study further compared the effects of three secretion elements, CysR, Amf, and Gla, on CHb production. RT-qPCR analysis revealed differences in steady-state CHb transcript abundance among the three secretion-element constructs. Because all three constructs employed the same *Ygpd* promoter and contained the same downstream *chb* coding sequence, these differences cannot simply be attributed to the promoter itself. Similar observations have been reported in other yeast expression systems. Ito et al. found that alterations to the MFα secretion leader in *P. pastoris* were accompanied by changes in target-gene mRNA abundance in some leader variants [18]. Xu et al. further showed that sequence composition in the 5′ coding region can affect transcript accumulation, potentially through effects on RNA stability or premature transcription termination [19]. Since CysR, Amf, and Gla form different 5′ coding-sequence contexts immediately upstream of *chb* in the respective fusion genes, these sequence differences provide a plausible explanation for at least part of the observed variation in steady-state CHb transcript abundance. Notably, steady-state CHb transcript abundance and total CHb production did not show a simple correspondence among the three secretion-element constructs. CysR-CHb exhibited substantially lower steady-state CHb transcript abundance than Amf-CHb and Gla-CHb, yet its total CHb production remained close to that of Amf-CHb and was higher than that of Gla-CHb. Conversely, Amf-CHb and Gla-CHb showed only a small difference in steady-state CHb transcript abundance, whereas their total CHb production differed markedly. These observations indicate that transcript abundance alone cannot fully account for the differences in CHb production among the three secretion-element constructs. More broadly, a large-scale quantitative transcriptomic and proteomic analysis of *S. cerevisiae* revealed a weak within-gene correlation between mRNA and corresponding protein abundances across natural isolates (median ρ = 0.165) [20]. Therefore, additional processes downstream of transcript accumulation, including translation, protein stability and turnover, intracellular trafficking, and secretion, may also contribute to the observed differences in CHb production, although these factors were not directly examined in the present study.

Among the three secretion elements tested, Amf resulted in the highest extracellular CHb production. This finding is consistent with observations in other yeast expression systems. Zou et al. systematically compared α-MF secretion leaders from different yeast species and multiple endogenous signal peptides for EGFP secretion in *P. pastoris*. The *S. cerevisiae* α-MF showed relatively high secretion performance, with only a small number of the tested α-MF leaders and endogenous signal peptides achieving comparable or higher levels of EGFP secretion. The study also showed that the performance of secretion elements is dependent on the target protein, indicating that the optimal secretion leader should be selected according to the specific recombinant protein [13]. Unlike CysR and Gla, which consist only of signal peptide sequences, Amf contains the complete MFα prepro leader, in which the pre-region mediates ER targeting and translocation, whereas the pro-region contributes additional functions in the secretory pathway [21]. This structural difference may contribute to the enhanced secretory performance of Amf. Direct experimental evidence supports the importance of the pro-region in recombinant protein secretion. In *Saccharomyces boulardii*, removal of the pro-region from the complete MFα prepro leader reduced extracellular NPA production from approximately 250 to 132 mg/L [22]. Similarly, in *S. cerevisiae*, inclusion of the complete α-factor pro-region markedly enhanced ER translocation and secretion of msGFP compared with the signal peptide alone, possibly by facilitating Kar2 (BiP)-mediated translocation and reducing polypeptide backsliding through the translocon [23]. N-linked glycosylation within the MFα pro-region may provide an additional mechanism contributing to its secretion-promoting function. Mutation of all three N-linked glycosylation sites in the α-factor pro-peptide reduced insulin precursor secretion to approximately 13% of that obtained with the fully glycosylated pro-peptide [24]. More recently, mutation of the three N-glycosylation sites in the *S. cerevisiae* α-MF leader was also shown to reduce EGFP secretion, whereas introduction of such sites into α-MF leaders lacking them enhanced secretion. The same study linked these effects to calnexin-associated protein folding and ER stress [25]. Taken together, these findings support the interpretation that the complete MFα pro-region is a likely contributor to the higher extracellular CHb production observed with Amf through its effects on ER translocation and N-glycosylation-associated protein folding and quality control, although these mechanisms remain to be directly verified in *T. fuciformis*. Interestingly, despite its substantially lower steady-state CHb transcript abundance, CysR-CHb still achieved a relatively high extracellular CHb yield, indicating that CysR can effectively mediate CHb secretion. Considering that Amf contains an additional pro-region with secretion-enhancing functions, whereas CysR consists solely of a signal peptide yet still mediates efficient CHb secretion, CysR may represent a promising endogenous secretion element for *T. fuciformis*. Future studies could further investigate its native leader sequence and evaluate its capacity to enhance the secretion of other heterologous proteins.

Reported production levels of recombinant hemoglobins in yeast span a wide range and are influenced by multiple factors, including the target protein, host background, intracellular or extracellular expression, and the extent of engineering [26]. The CHb examined in the present study has previously been produced intracellularly in *S. cerevisiae*, reaching 20.7 mg/L during shake-flask expression screening [15]. For the secretory production of other plant hemoglobins, Bae et al. achieved extracellular gmaLegHb titers of 398.1 and 1652.7 mg/L in *S. cerevisiae* and *P. pastoris*, respectively, through signal peptide screening and hemin supplementation [27]. Previous research has further shown that, through multilevel optimization strategies, including gene dosage optimization, engineering of the heme biosynthetic pathway, and 10-L high-density fed-batch fermentation, the secreted LegH titer in *P. pastoris* reached 3.5 g/L [28]. Therefore, the estimated extracellular CHb yield of 106.43 mg/L obtained in this study indicates that *T. fuciformis* YLCs can support extracellular production of the recombinant CHb construct at the 100 mg/L scale, although the proportion present as holo-CHb was not determined. Previous quantitative studies of other heterologous proteins in *T. fuciformis* have generally reported production levels in the μg/g to sub-mg/g dry weight (DW) range. For example, hepatitis B surface antigen (HBsAg) was reported at 0.4–1.4 μg/g DW in *T. fuciformis* YLCs [3], whereas the hydrophobins Po.HYD from *Pleurotus ostreatus* and Ltr.HYD from *Lentinus tuber-regium* were produced at 0.58 and 0.66 mg/g DW, respectively [4,29]. In contrast, a recent study employed a more efficient endogenous promoter together with signal peptide replacement and codon optimization, resulting in an average production level of 20.60 mg/g DW for *P. ostreatus* hydrophobin Vmh2 and its mutants in *T. fuciformis* [30]. Beyond optimization of the expression and secretion systems, heme supply represents another targeted strategy for improving hemoglobin production. Previous research has shown that, following optimization of globin expression, further enhancement of heme biosynthesis can still substantially improve hemoglobin production. In *P. pastoris*, combinatorial overexpression of multiple *HEM* genes in the heme biosynthetic pathway increased the secreted LegH titer at this engineering stage to 529 mg/L, approximately 2.1 times the level before heme pathway optimization, while its peroxidase activity increased to 9.0 times the pre-optimization level [28]. Taken together, future improvements in CHb production could involve promoter engineering, secretion-element optimization, and further coding-sequence optimization, together with engineering of the heme biosynthetic pathway, thereby targeting multiple levels of transcription, translation, secretion, and hemoglobin maturation. Further optimization of culture conditions and fermentation processes may also improve CHb production during scale-up. Because these aspects have not yet been systematically optimized, the CHb production level achieved in the present study should not be regarded as the upper limit of the production capacity of the *T. fuciformis* expression system.

By comparison, *S. cerevisiae* has a well-characterized genetic background and a broad range of molecular genetic tools, and multilevel strategies have been established to optimize heterologous protein production, including CRISPR/Cas9 genome editing, promoter and terminator engineering, gene copy-number regulation, secretion pathway engineering, and systems-level metabolic engineering [31]. *P. pastoris* likewise possesses well-developed tools for genome editing and expression regulation, including CRISPR/Cas systems, inducible and constitutive promoters, gene dosage regulation, and engineering of protein folding and secretion pathways. In addition, this host has well-established high-cell-density cultivation and large-scale fermentation systems [32]. Filamentous fungi are also important hosts for industrial protein production because of their strong natural protein secretion capacity and eukaryotic protein-processing machinery, and species of *Aspergillus*, *Trichoderma*, and *Penicillium* have been widely used for the production of industrial enzymes and heterologous proteins [33]. However, the complex mycelial morphologies resulting from filamentous growth can markedly affect broth rheology, mixing, and oxygen and nutrient transfer, thereby increasing the complexity of process control and scale-up in submerged fermentation [34]. In *T. fuciformis*, basidiospores can form YLCs through budding, and these cells can be cultivated in liquid media and have been used for the expression of various heterologous proteins. Its edible fungal origin, yeast-like growth form, and eukaryotic protein-processing capacity support its development as a recombinant protein host that combines yeast-like liquid cultivation with eukaryotic protein-processing capability. However, compared with well-established yeast expression hosts, the *T. fuciformis* expression system remains at a relatively early stage of development, with a relatively limited repertoire of efficient endogenous expression-regulatory elements currently available, while its genetic transformation methods still require further refinement [35]. During scale-up from laboratory to industrial production, oxygen transfer, mixing, and environmental heterogeneity in large-scale bioreactors may affect cell growth, production efficiency, and product quality. Therefore, the robustness of CHb production during scale-up needs to be further evaluated, together with optimization of the fermentation process [36]. In the present study, extracellular secretion of CHb reduced the need for cell disruption, thereby simplifying an important step in downstream recovery; nevertheless, extracellular production still requires appropriate processes for cell removal, concentration, and purification, and the recovery efficiency, purity, and downstream processing costs remain to be systematically evaluated [37]. Furthermore, the industrial feasibility of food precision fermentation is influenced by multiple cost-related factors, including cultivation and energy inputs, process design, and downstream processing [38], whereas medium and feed costs, the unit production cost of CHb, and the overall process economics were not systematically assessed in the present study.

Building on previous studies of heterologous protein expression in *T. fuciformis*, the present study combined the comparison of candidate hemoglobins with subsequent secretion-element screening within the same *T. fuciformis* YLC expression platform, thereby evaluating recombinant hemoglobin production from the perspectives of both target-protein selection and secretion-strategy optimization. Through this stepwise screening approach, CHb and Amf were identified as the best-performing hemoglobin and secretion element, respectively, among those tested. Extracellular CHb production with the CHb–Amf combination reached the 100 mg/L scale, providing a quantitative basis for further development of *T. fuciformis* as a host for recombinant globin production. Direct characterization of heme incorporation and protein function will be required in future studies.

## 5. Conclusions

This study evaluated *T. fuciformis* YLCs as a host system for recombinant hemoglobin production. Among the three candidate hemoglobins, CHb showed the highest observed estimated protein yield and was therefore selected for subsequent secretion element screening. All three tested secretion elements mediated CHb secretion, with Amf showing the best overall performance and achieving 106.43 mg/L extracellular CHb and 133.02 mg/L total CHb production. These results identified CHb and Amf as a suitable target protein and secretion element combination for recombinant hemoglobin production in *T. fuciformis* YLCs. The findings support further development of *T. fuciformis* YLC-based expression systems for food-related recombinant protein production. However, direct characterization of heme incorporation and functional properties of the recombinant CHb will be required before its suitability for specific food applications can be established.

## Figures and Tables

**Table 1:** Primers Used for Polymerase Chain Reaction (PCR) and Reverse Transcription Quantitative PCR (RT-qPCR) Analysis.

Primer	Sequence (5′ → 3′)	Purpose
*Ygpd*-F	aagcttCTCGCTGCACGCTTCTCGAC	Amplification of the *Ygpd* promoter
*Ygpd*-R	acgcgtTTGCGCCATACGTTGGATGCG	
18-T-*Mlu*I-*chb*-F	ACGTATGGCGCAAGGATCCCGATCTacgcgtATGGCGTTCACGGACAAGCAG	Amplification of *chb* for vector construction
18-T-*Hin*dIII-*chb*-R	ATGATTACGAATTCGAGCTCGGTACaagcttTTAGTGGTGGTGGTGGTGGTGC	
18-T-*Mlu*I-*vgb*-F	ACGTATGGCGCAAGGATCCCGATCTacgcgtATGCTTGACCAGCAGACGATCAACA	Amplification of *vgb* for vector construction
18-T-*Hin*dIII-*vgb*-R	ATGATTACGAATTCGAGCTCGGTACaagcttttaGTGGTGGTGGTGGTGGTGTTC	
18-T-*Mlu*I-*lb2*-F	ACGTATGGCGCAAGGATCCCGATCTacgcgtATGGTCGCGTTCTCGGACAAG	Amplification of *lb2* for vector construction
18-T-*Hin*dIII-*lb2*-R	ATGATTACGAATTCGAGCTCGGTACaagcttTTAGTGGTGGTGGTGGTGGTGGTA	
18-T-*Hin*dIII-*Ygpd*-F	CCaagcttCTCGCTGCACGCTTCT	Amplification of the *Ygpd*-Hb expression cassette
His-*Kpn*I-R	GGggtaccTTAGTGGTGGTGGTGGTGG	
18-T-*Mlu*I-cysr-F	ACGTATGGCGCAAGGATCCCGATCTacgcgtATGCTTTCGCTTCACCTTGTTTCGC	Amplification of the CysR-chb fusion fragment (paired with 18-T-*Hin*dIII-chb-R)
18-T-*Mlu*I-amf-F	ACGTATGGCGCAAGGATCCC	Amplification of the Amf-vgb fusion fragment (paired with 18-T-*Hin*dIII-vgb-R)
18-T-*Mlu*I-gla-F	ACGTATGGCGCAAGGATCCCGATCTacgcgtATGTCGTTCCGATCTCTACTCGC	Amplification of the Gla-lb2 fusion fragment (paired with 18-T-*Hin*dIII-lb2-R)
Primer	Sequence (5′ → 3′)	Purpose
*chb*-F(amf-*chb*)	GAAAAGAGAGGCTGAAGCTgggcccATGGCGTTCACGGACAAGCA	Amplification of *chb* for In-Fusion replacement of *vgb* in pGEH-Ygpd-Amf-vgb (paired with gene-R)
*chb*-F(gla-*chb*)	AAATGTGATTTCGAAGCGCgggcccATGGCGTTCACGGACAAGCA	Amplification of *chb* for In-Fusion replacement of *lb2* in pGEH-Ygpd-Gla-lb2 (paired with gene-R)
gene-R	GCAGCGAGAATTCGAGCTCG	Common reverse primer for *chb* amplification during In-Fusion-mediated gene replacement
*chb*-*gpd*-F	GACAAGGCCGAAGACCTTTTCC	Forward primer for colony PCR screening of pGEH-Ygpd-chb, pGEH-Ygpd-CysR-chb, and pGEH-Ygpd-Gla-chb, paired with pgeh-gpd-R
*vhb*-*gpd*-F	CCAAGACTGTCATAGCAAGAGCCT	Forward primer for colony PCR screening of pGEH-Ygpd-vgb, paired with pgeh-gpd-R
*lb2*-*gpd*-F	TGAGCTTAGGGTTCGTCGCA	Forward primer for colony PCR screening of pGEH-Ygpd-lb2, paired with pgeh-gpd-R
Amf-*gpd*-F	ATCCCCTTCTAAATCTGAGTAACCGATG	Forward primer for colony PCR screening of pGEH-Ygpd-Amf-chb, paired with pgeh-gpd-R
pgeh-*gpd*-R	GACAAGTGTGTCGTGCTCCAC	Common reverse primer for colony PCR screening of recombinant pGEH expression vectors
EH-F	GCAGAAGAACGGCATCAAGGTG	PCR verification of transformants
EH-R	CAGGCTCTCGCTAAACTCCCC	
*β-tubulin*-F	GATGACCATTTCTTGCTTC	Reference gene for RT-qPCR
*β-tubulin*-R	GTTCTGACATTTGCTACCG	
*chb*-F	AGGAGAAAAGTTCCTTAGCAGCG	RT-qPCR analysis of *chb* expression
*chb*-R	GAATCCCTTGTCAACTCGTCGTA	
*vgb*-F	GCTACAGTTCCAGTTCTCAAGGA	RT-qPCR analysis of *vgb* expression
*vgb*-R	ACTGTCATAGCAAGAGCCTTAGG	
*lb2*-F	GTCTTCTACACGACGATCCTTGA	RT-qPCR analysis of *lb2* expression
*lb2*-R	CTGACAAGACCGAAGAGCTTTTC	

## Data Availability

The original contributions presented in this study are included in the article/Supplementary Materials. Further inquiries can be directed to the corresponding authors.

## Supplementary Materials

The following supporting information can be downloaded at: https://www.mdpi.com/article/10.3390/foods15173026/s1, Figure S1: SDS-PAGE analysis of culture supernatants from the VHb-, CHb-, and Lb2-expressing transformants lacking secretion elements.

## Abbreviations

Abbreviation	Full term
ALA	5-Aminolevulinic acid
Amf	α-mating factor prepro leader
BCA	Bicinchoninic acid
CFU	Colony-forming units
CHb	Clover hemoglobin
CTAB	Cetyltrimethylammonium bromide
CysR	Cysteine-rich protein-derived signal peptide
ΔCt	Delta threshold cycle
eGFP	Enhanced green fluorescent protein
ER	Endoplasmic reticulum
Gla	Glucoamylase signal peptide
gmaLegHb	*Glycine max* leghemoglobin
Hb	Hemoglobin
IM	Induction medium
LB	Luria–Bertani
Lb2	*Vigna unguiculata* leghemoglobin 2
LegH	Leghemoglobin
MFα	α-Mating factor
msGFP	Monomeric superfolder green fluorescent protein
NPA	*Clostridioides difficile* Toxin A neutralizing peptide
NTC	No-template control
OD_600_	Optical density at 600 nm
PCR	Polymerase chain reaction
PDA	Potato dextrose agar
RT-qPCR	Reverse transcription quantitative PCR
SD	Standard deviation
SDS-PAGE	Sodium dodecyl sulfate–polyacrylamide gel electrophoresis
TCA	Trichloroacetic acid
VHb	*Vitreoscilla* hemoglobin
WT	Wild type
*Ygpd*	*T. fuciformis* glyceraldehyde-3-phosphate dehydrogenase promoter
YLCs	Yeast-like cells
