# Supplementary material for: Production of Recombinant Hemoglobin in Tremella fuciformis Yeast-like Cells Through Selection of Hemoglobins and Secretion Elements
Source: Foods. 2026 Aug 27;15(17):3026. doi: 10.3390/foods15173026 (PMC13565620; doi:10.3390/foods15173026)
Supplement: Supplementary file 1 [file foods-15-03026-s001.zip › foods-4459172-supplementary.pdf]

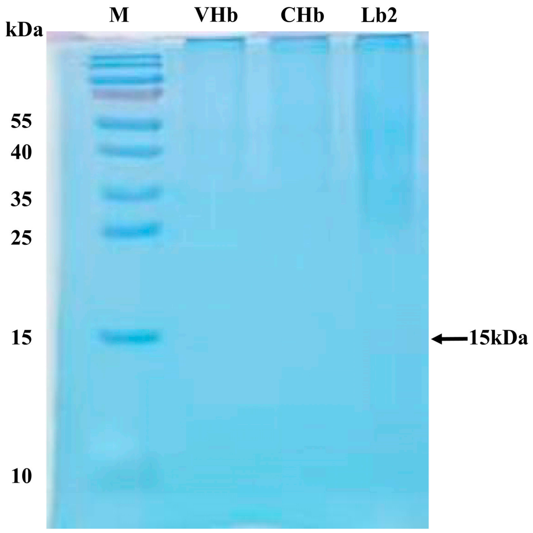

Figure S1. SDS-PAGE analysis of culture supernatants from the VHb-, CHb-, and Lb2-expressing transformants lacking secretion elements. Lane M, protein molecular weight marker; lane VHb, VHb-only transformant; lane CHb, CHb-only transformant; lane Lb2, Lb2-only transformant. No detectable protein band corresponding to the expected molecular weight of CHb (~15 kDa) was observed in the CHb lane under the same analytical conditions used for the secretion-element transformants.
